# HCMV Infection and Apoptosis: How Do Monocytes Survive HCMV Infection?

**DOI:** 10.3390/v10100533

**Published:** 2018-09-29

**Authors:** Donna Collins-McMillen, Liudmila Chesnokova, Byeong-Jae Lee, Heather L. Fulkerson, Reynell Brooks, Bailey S. Mosher, Andrew D. Yurochko

**Affiliations:** 1 BIO5 Institute, Department of Immunology, University of Arizona, Tucson, AZ 85721, USA; dcoll2@email.arizona.edu; 2 Department of Microbiology & Immunology, Center for Molecular & Tumor Virology, Louisiana State University Health Sciences Center—Shreveport, Shreveport, LA 71130, USA; lchesn@lsuhsc.edu (L.C.); blee19@lsuhsc.edu (B.-J.L.); hfulke@lsuhsc.edu (H.L.F.); reynellbrooks@gmail.com (R.B.); bsnave@lsuhsc.edu (B.S.M.); 3 Center for Cardiovascular Diseases & Sciences, Louisiana State University Health Sciences Center—Shreveport, Shreveport, LA 71130, USA; 4 Feist-Weiller Cancer Center, Louisiana State University Health Sciences Center—Shreveport, Shreveport, LA 71130, USA; 5 Center of Excellence in Arthritis & Rheumatology, Louisiana State University Health Sciences Center—Shreveport, Shreveport, LA 71130, USA

**Keywords:** human cytomegalovirus, apoptosis, programmed cell death, cell signaling, monocytes, macrophages, survival, differentiation

## Abstract

Human cytomegalovirus (HCMV) infection of peripheral blood monocytes plays a key role in the hematogenous dissemination of the virus to multiple organ systems following primary infection or reactivation of latent virus in the bone marrow. Monocytes have a short life span of 1–3 days in circulation; thus, HCMV must alter their survival and differentiation to utilize these cells and their differentiated counterparts—macrophages—for dissemination and long term viral persistence. Because monocytes are not initially permissive for viral gene expression and replication, HCMV must control host-derived factors early during infection to prevent apoptosis or programmed cell death prior to viral induced differentiation into naturally long-lived macrophages. This review provides a short overview of HCMV infection of monocytes and describes how HCMV has evolved to utilize host cell anti-apoptotic pathways to allow infected monocytes to bridge the 48–72 h viability gate so that differentiation into a long term stable mature cell can occur. Because viral gene expression is delayed in monocytes following initial infection and only occurs (begins around two to three weeks post infection in our model) following what appears to be complete differentiation into mature macrophages or dendritic cells, or both; virally-encoded anti-apoptotic gene products cannot initially control long term infected cell survival. Anti-apoptotic viral genes are discussed in the second section of this review and we argue they would play an important role in long term macrophage or dendritic cell survival following infection-induced differentiation.

## 1. Introduction

Human cytomegalovirus (HCMV) infection can cause significant disease in immunocompromised and immunocompetent hosts [1,2,3]. In the immunocompromised, HCMV infection causes severe and often fatal disease in transplant recipients and in congenitally infected neonates [1,2,3]. In the immunocompetent, HCMV infection can cause mononucleosis and is associated with a variety of chronic cardiovascular diseases [1,2,3]. HCMV has a broad cellular tropism and can infect a variety of cell types in vivo and in vitro [4]. This broad tropism allows the virus to infect most host organ systems, which in turn can cause the overt organ disease observed.

On a cellular level, the outcome of infection varies widely according to the cell type infected, and in some cases, whether a primary infection or a latent infection occurs. For example, cells such as fibroblasts and smooth muscle cells are primary targets for HCMV replication and provide a platform from which large quantities of viral progeny can be made [4]. Other cells, such as epithelial and endothelial cells, support lower levels of viral replication and instead undergo a chronic or “smoldering” infection from which low levels of persistent viral shedding presumably occur [4]. This is certainly consistent with the long-term viral shedding, for example, seen in the urine of congenitally infected children and in the breast milk of women post birth [1,5,6,7]. Hematopoietic cells such as CD34+ human progenitor cells (HPCs) and their more differentiated counterparts—monocytes—do not support a true replicative infection (at least not initially [8,9,10,11,12,13,14,15]). CD34+ HPCs serve as a reservoir for viral latency in the bone marrow (thus expressing only select latent transcripts) [14,16] and following appropriate stimuli they also serve to seed infected monocytes into the peripheral blood [17,18]. Blood monocytes thus serve as essential vehicles for hematogenous dissemination following reactivation and differentiation of CD34+ HPCs into monocytes that in turn allow organ dissemination following migration and differentiation of these cells into tissue macrophages or dendritic cells [19,20,21,22,23,24,25]. In addition, and key to this review is that following primary infection, infected blood monocytes (see below for more detail) serve as primary vehicles or Trojan horses for dissemination of the virus to most organ systems allowing the initial infection to proceed and the establishment of life-long persistence and latency. When these cells differentiate along the myeloid lineage into mature macrophages or dendritic cells, viral gene expression is triggered and viral progeny are produced [21,23,24,25,26]. Neutrophils have also been implicated in hematogenous dissemination of HCMV, and like monocytes and CD34+ HPCs, do not support a productive (replicative) infection [27]. However, unlike CD34+ HPCs and monocytes—which differentiate and thus maintain the virus in their differentiated lineages and eventually become a source of long term mature virus production—neutrophils only appear to serve as vehicles for dissemination from blood to organ tissue [27].

As discussed above, the hematogenous spread of HCMV following primary infection to host organs occurs in a cell dependent manner and monocytes play a key, if not essential, role [28,29,30]. HCMV DNA and proteins can be detected in monocytes during acute infection [1,4,31] and they are the predominant infiltrating cell type found in infected organs [1,32,33]. Nevertheless, although monocytes are infected (virus enters these cells) [34,35,36,37] and are in “the right place at the right time” for hematogenous dissemination of the virus, they are not permissive for viral replication [8,9,10,11,12,13,14,15,26,38,39,40]. The short life span of monocytes (1–3 days) [41] is also an undesirable characteristic for HCMV because of its slow replication cycle of days to weeks in vivo [1]. Macrophages, on the other hand, are naturally long-lived cells (months to possibly years), are productively infected by HCMV in vivo, and are sites of persistent viral release in organ tissue [23,26,38,39,40], and cannot be a source of viral spread from the blood because they are not blood-borne cells [41]. In concert, monocytes and macrophages thus possess all the necessary features to promote viral spread to, and the establishment of, persistence in host organ systems.

**Figure 1:**
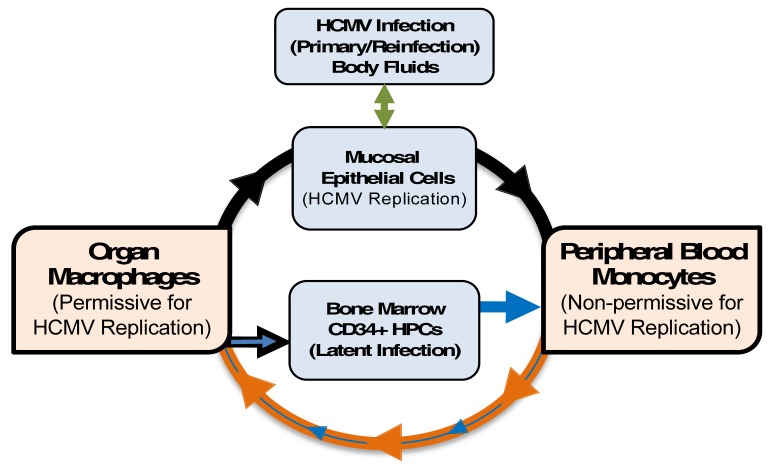
Model: Monocytes are a primary source of viral dissemination and persistence. Initially, epithelial cells (green arrow) of a naïve host are infected by virus shed in bodily fluids such as saliva, urine, or breast milk from an infected host [4]. Infection then spreads to the blood (black arrow) via an unclear mechanism (perhaps by dendritic cells) [42] and infects (at least enters) blood monocytes [1,4,43]. Next, infected monocytes extravasate, carrying the infectious virus, to peripheral tissue. The extravasation (and cellular activation) would primarily be mediated by activation of monocytes due to viral envelope (and possibly tegument) products, independent of new viral gene expression [30,34,36,37,44,45,46]. Infected monocytes would then differentiate (purple arrow) into naturally long-lived tissue macrophages [30,47] that are permissive for viral replication [1,4,23,26,38], allowing for replication of the original infectious virus carried into the tissue by monocytes [30,34,37,48]. This would ensure persistence within the host through chronic release of virus from macrophages (consistent with data from patients) [1], and the establishment of latency through the migration of infected blood monocytes into the bone marrow [22,23,49,50]. Reinfection of epithelial cells (black arrow) at “portals of virus exit” would allow spread to naïve hosts (green arrow). This process of viral spread utilizing myeloid cells initially occurs following primary infection. Reactivation of latent virus from CD34+ human progenitor cells (HPCs) (yellow/gold arrow) occurs throughout the life of the host, and thus serves as a long-term source of infected monocytes. Because these monocytes are latently infected (small yellow/gold arrow), one would expect aspects of the molecular consequences of latent infection of monocytes to be distinct from that seen following primary infection of monocytes. HCMV: human cytomegalovirus.

This review discusses our model system of using primary human blood monocytes isolated from healthy human donors to mimic a primary infection. Specifically, we have focused on hematogenous dissemination as would occur following recruitment of peripheral blood monocytes to a site of replicative infection (e.g., initially infected oral epithelial cells of a host; see Figure 1, which represents our model) and then spread of the infected leukocyte to organ tissue. Although we cannot account for signaling, such as may occur in other peripheral blood cells in close proximity or in the surrounding tissues, our model allows us to examine the effects of HCMV infection on cellular signaling in healthy primary human cells. Under these conditions, we do not detect de novo viral gene expression in monocytes until approximately two to three weeks post infection when monocyte-to-macrophage differentiation is complete [30], consistent with reports from other laboratories [23,24,25]. A major focus of our work has been determining how, in the absence of viral gene expression, HCMV activates the infected monocyte and alters the molecular processes that promote survival of infected monocytes despite their biologically limited lifespan [41] and the induction of a pro-apoptotic cellular defense and then promotes their differentiation into naturally long-lived macrophages [48]. Importantly, we have shown that viral anti-apoptotic transcripts are not expressed during at least the first 72 h following infection of primary monocytes [51], consistent with the delayed de-envelopment and nuclear translocation [35] and the delayed onset of viral gene expression until differentiation is completed [30]. Instead, early survival of HCMV-infected monocytes is mediated in part by viral-induced regulation of the cellular myeloid cell leukemia-1 (Mcl-1) and B cell lymphoma-2 (Bcl-2) anti-apoptotic proteins [48,51,52]. Our recent findings in this area are summarized in the first section of this review. Once infected monocytes have differentiated into mature macrophages/dendritic cells—a process that is driven by HCMV infection [47,48,53,54]—viral gene products are made [30,48] and can presumably function to promote continued survival of the infected cell. Although a number of other cell death pathways are likely activated during HCMV infection, we have chosen to focus on viral manipulation of apoptotic pathways for this review, as our studies performed in quiescently-infected monocytes have centered around up-regulation of cellular anti-apoptotic factors. The second section of this review includes a brief summary of the anti-apoptotic proteins known to be encoded by HCMV and describes their specific functions in promoting survival of the infected cell.

Apoptosis is a form of programmed cell death that is characterized by a reduction in cellular and nuclear volume, nuclear fragmentation with only minor modification of cytoplasmic organelles, plasma membrane blebbing, and engulfment by phagocytic cells [55]. On the biochemical level, apoptosis is associated with exposure of phosphatidylserine on the outer layer of the plasma membrane, degradation of nuclear DNA, and the loss of mitochondrial transmembrane potential. This process is mediated by the sequential proteolytic cleavage and activation of caspases, a family of cysteine proteases which target aspartate residues in their substrates [56,57]. The DNA fragmentation which is characteristic of apoptotic cell death is mediated directly by three effector caspases: caspase-3, caspase-6, and caspase-7 [58,59]. Apoptosis can be broadly classified as either extrinsic, which is initiated primarily in response to extracellular cues and occurs through activation of transmembrane receptors, or intrinsic, which occurs as a result of perturbations in the cellular microenvironment and is controlled by Bcl-2 family proteins at the mitochondria [60]. Although the extrinsic and intrinsic apoptotic pathways can be independently defined at the molecular level, there is substantial evidence of crosstalk among the two pathways as well [61]. Importantly, both extrinsic and intrinsic apoptotic pathways can be triggered in response to activation of Toll-like receptors (TLRs) as a result of pathogen infection [62].

Extrinsic apoptosis occurs following transmission of a death signal from the extracellular environment into the cell. The death signal can be transmitted by two distinct types of plasma membrane receptors. The first type is death receptors, which are activated in response to binding by their cognate ligands (e.g., Fas ligand (FasL)/Fas receptor (FasR), tumor necrosis factor α (TNF-α)/tumor necrosis factor α receptor 1 (TNFR1), Apo3L/DR3, Apo2L/DR4, and Apo2L/DR5) [63,64,65,66,67,68]. Activation of these receptors results in assembly of a multi-protein complex (e.g., death-inducing signaling complex or DISC) which regulates the cleavage and activation of initiator caspases (caspase-8 and caspase-10) [69,70]. The fully active initiator caspases then drive proteolytic cleavage and activation of effector caspases (caspase-3 and caspase-7) [57,71]. The second type of plasma membrane receptor involved in triggering extrinsic apoptosis is the family of dependence receptors, which are activated when their specific ligands drop below a normal physiological threshold (e.g., the deleted in colorectal carcinoma (DCC) netrin 1 receptor, the unc-5 netrin receptor A (UNC5A), the sonic hedgehog receptor patched 1 (PTCH1), Plexin D1, the nerve growth factor receptor (p75NGFR), and the insulin-like growth factor receptor 1 (IGF-1 receptor) [72,73,74,75,76,77]. Under normal homeostatic conditions, the dependence receptors promote cell survival; however, they activate signaling cascades resulting in caspase activation when their cognate receptors fall below the normal physiological threshold [78].

Intrinsic apoptosis occurs in response to physiological imbalances within the cell and can be triggered by DNA damage, growth factor withdrawal, accumulation of unfolded proteins in the endoplasmic reticulum (ER), reactive oxygen species (ROS) overload, or in response to pathogen infection. Activation of caspases in the intrinsic pathway is controlled at the mitochondrial membrane by members of the Bcl-2 family of proteins [79,80,81,82]. These proteins can be classified into three distinct subgroups: pro-apoptotic, anti-apoptotic, and BH3 only proteins [83]. As their name suggests, pro-apoptotic Bcl-2 family members (e.g., Bax and Bak) are directly responsible for triggering apoptosis. These proteins form dimers that open channels in the mitochondrial membrane, releasing cytochrome C and other pro-apoptotic factors into the cytoplasm and beginning the caspase activation cascade [83]. During intrinsic apoptosis, the initiator caspase-9 is activated first, followed by activation of caspase-3 and caspase-7 [57,84,85]. The formation of pro-apoptotic dimers can be prevented via binding of anti-apoptotic Bcl-2 family proteins (e.g., Mcl-1 and Bcl-2) to the pro-apoptotic proteins; thus, the ratio of anti-apoptotic to pro-apoptotic proteins in a given cell determines its apoptotic fate [83]. An additional level of regulation exists in the BH3 only proteins (e.g., Bid, Bim, Puma, Noxa). These proteins are indirectly pro-apoptotic, as their binding to anti-apoptotic proteins prevents binding of the anti-apoptotic proteins by pro-apoptotic proteins [83]. This interaction allows dimerization of the pro-apoptotic proteins and mitochondrial outer membrane permeabilization (MOMP) [85].

## 2. Cellular Proteins

**Figure 2:**
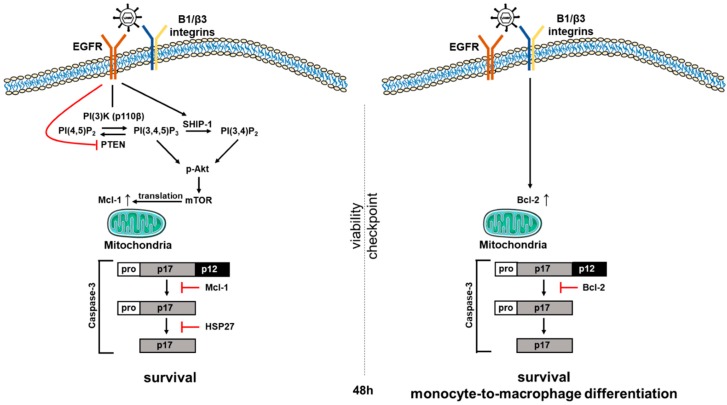
HCMV-induced signaling induces an early biphasic pro-survival state in infected monocytes. We have shown that viral binding to epidermal growth factor receptor (EGFR) and integrins on the surface of target monocytes initiates a unique signaling cascade that alters the expression of multiple Bcl-2 family proteins in infected monocytes over time. Mcl-1 levels are enhanced via EGFR activation for the first 48 h post infection (hpi), allowing infected cells to successfully navigate the early 48-h (48 h) cell viability checkpoint. siRNA knockdown supports the critical role for Mcl-1 in monocyte survival early after infection. Mcl-1, in cooperation with HSP27, blocks the proteolytic cleavage and activation of caspase-3, ensuring early survival of infected monocytes. Once infected cells survive to and through the 48 h viability gate, the loss of Mcl-1 allows a basal activation of the caspase-3 required for differentiation. In addition, HCMV-induced integrin signaling that initially was stimulated during viral binding induces a delayed Bcl-2 expression by 48 hpi to sustain the pro-survival phenotype beyond the 48 h viability gate, while allowing low level activation of caspase-3 to continue to promote the monocyte-to-macrophage differentiation required for full viral permissiveness in these differentiated cells. siRNA knockdown supports the critical role for Bcl-2 in monocyte survival 48 h post HCMV infection.

In non-permissive cells or in cells with a delayed life cycle (i.e., delayed gene expression), virally encoded anti-apoptotic factors would not be expected to play a prominent role early in cell survival, thus HCMV must regulate the apoptotic process in these cells by an alternative mechanism. Our work has revealed one such viral-regulated pro-survival mechanism that can function in the absence of de novo viral gene expression. We show that during infection of non-permissive monocytes, HCMV usurps cellular signaling pathways through receptor-ligand engagement to alter the expression pattern of cellular anti-apoptotic proteins [47,51,52,86] (Figure 2). In this scenario, the viral glycoproteins (glycoprotein B (gB) and the gH/gL complexes (the trimer and the pentamer)) bind to their cognate receptors, the epidermal growth factor receptor (EGFR) and the β1/β3-integrins, respectively, to promote cell survival through the targeted transcription of cellular anti-apoptotic factors such as Mcl-1 [51] and Bcl-2 [52]. Viral regulation of Mcl-1 and Bcl-2 expression allows for strict control over caspase function during infection, allowing the virus to not only control cell survival, but also direct monocyte-to-macrophage differentiation [47].

### 2.1. Mcl-1

HCMV infection of monocytes is critical for the hematogenous dissemination of the virus throughout the host following primary infection (reviewed in Reference [28]). To this end, HCMV must overcome several biological barriers that typically limit monocyte infection. One such biological barrier is the short lifespan of blood monocytes. Upon entry into circulation, monocytes live for 48–72 h before undergoing apoptosis unless the monocyte differentiates into a long-lived macrophage [87]. HCMV evolved to extend the viability of infected monocytes by preventing apoptosis, thus allowing differentiation into productive macrophages. To block apoptosis early during infection and bypass the initial 48-h (48 h in Figure 2) viability checkpoint, HCMV alters the expression of the cellular anti-apoptotic protein Mcl-1.

Mcl-1 is a member of the anti-apoptotic Bcl-2 family of proteins. Characteristic of this family of proteins, Mcl-1 contains multiple Bcl-2 homology (BH) domains that allow for heterodimerization with other Bcl-2 family proteins. The hydrophobic BH3 domain-binding pocket of the anti-apoptotic proteins (i.e., Bcl-2 and Mcl-1) allows for binding to pro-apoptotic Bcl-2 family members (i.e., Bak and Bax), which inhibits apoptosis. This hydrophobic BH3 domain-binding pocket specifies to which pro-apoptotic proteins each anti-apoptotic protein can bind. Mcl-1 can bind BH3 only proteins such as Bim, Bid, Puma, and Noxa, as well as the pro-apoptotic effector Bak. Mcl-1 can therefore prevent Bak oligomerization at the mitochondrial outer membrane in two ways: (1) sequestering BH3 only pro-apoptotic proteins, thus preventing activation of Bak, and (2) sequestering Bak to prevent activation by the BH3 only proteins. Preventing Bak oligomerization inhibits perforation of the mitochondrial outer membrane, the subsequent release of cytochrome C and caspase activation, and thus inhibits apoptosis [88,89].

Our lab demonstrated that the first step in the HCMV-mediated inhibition of monocyte apoptosis occurs within the first 48 h post infection (hpi) and depends upon the up-regulation and stabilization of Mcl-1 [51]. The binding of HCMV gB to cellular EGFR, as well as the binding of the pentameric complex gH/gL/UL128-131 to β1 and β3 integrins drives a signaling pathway through PI(3)K (phosphatidylinositol-3-kinase) and induces an extended non-canonical activation of Akt [90] that leads to the phosphorylation of the mTOR kinase. The HCMV-induced phosphorylation of mTOR kinase leads to the up-regulation of Mcl-1 and heat shock protein 27 (HSP27) translation [51,91]. HCMV infection of monocytes leads to the up-regulation of Mcl-1 for up to 24 hpi at which point the levels of Mcl-1 begin to slowly decrease until reaching levels of mock-infected monocytes at 72 hpi. Mock-infected monocytes initially have high levels of Mcl-1 for up to 24 h post-harvest, at which time Mcl-1 levels diminish rapidly. siRNA knock down of Mcl-1 in both mock-infected and HCMV-infected monocytes resulted in decreased cell survival. Upon Mcl-1 knockdown, there was a 3-fold increase in apoptosis in mock-infected monocytes and a 5-fold increase in apoptosis in HCMV-infected monocytes [51]. These data suggest that Mcl-1 regulates early survival of HCMV-infected monocytes and also plays a role in the survival of uninfected monocytes. Further investigation into how HCMV up-regulation of Mcl-1 inhibits apoptosis revealed that Mcl-1 and HSP27 block the cleavage of caspase-3, which will be discussed in greater detail below [47]. Further support for the role Mcl-1 plays during early infection of monocytes comes from Reeves et al. [92] who showed that the Mcl-1 upregulated via gB and MAPK signaling in the absence of de novo viral gene products was important for myeloid cell survival in a model of experimental latency.

### 2.2. Bcl-2

While extended regulation of Mcl-1 promotes the early anti-apoptotic events for up to 48 hpi, thus quickly protecting the infected monocyte, the virus also induces a shift from Mcl-1 to Bcl-2 as the primary anti-apoptotic promoter of monocyte survival beyond 48 hpi [52]. This upregulation of Bcl-2 is mediated by distinct integrin signaling events following initial viral binding that induce HCMV-infected monocytes to exhibit an increase in Bcl-2 mRNA transcription at approximately 24 hpi; Bcl-2 protein levels are increased around 48 hpi. The increase in Bcl-2 occurs largely in response to the up-regulation of pro-apoptotic Bax following infection, which can be inactivated by Bcl-2, but not Mcl-1. The ratio of anti-apoptotic proteins, such as Bcl-2, versus pro-apoptotic proteins, such as Bax, determines the programmed fate of the cell. When expression of anti-apoptotic proteins is higher than that of pro-apoptotic proteins, cell survival is maintained. Thus, when HCMV induces an increase in Bcl-2 expression, the ratio is tipped in favor of cell survival, as excess Bcl-2 is able to bind and inactivate Bax, preventing its dimerization and insertion into the mitochondrial membrane and blocking subsequent channel formation that allows release of cytochrome C and activation of the caspase cascade. As this overlaps with the decrease in Mcl-1 at 48 hpi in HCMV-infected monocytes, Bcl-2 appears to assume the anti-apoptotic role of Mcl-1 and exhibits a dominant role in the long-term survival of the infected monocyte after the initial viability checkpoint has been navigated. Importantly, pre-treatment with the Bcl-2-specific small molecule inhibitor ABT-199 results in a significant decrease in the percentage of cell survival beginning at 24 hpi in uninfected cells and at 48 hpi in HCMV-infected cells. This virally induced bi-phasic shift in dominance of anti-apoptotic proteins pushes the infected circulating monocyte beyond the innate programming of cell death within 48–72 h, facilitating viral dissemination throughout the body [52]. This regulation in infected cells is distinct and in contrast with the regulation of these proteins in uninfected cells, where they are used together to navigate apoptotic programming, rather than in a linear or temporally individual fashion or in LPS-treated cells where Bcl-2/Bax interactions do not appear to control infected cell survival [52].

### 2.3. Caspase-3

Caspases are a family of genes important for maintaining cellular homeostasis through the regulation of cell death and inflammation [56,93]. The gene products hydrolyze peptide bonds to generate active signaling molecules that participate in ordered processes such as programmed cell death. Caspases are initially produced as inactive procaspases that require dimerization and often cleavage by adaptor proteins to become activated. Caspase-3 is a cysteine protease that functions as an executioner caspase by cleaving cellular DNA and proteins that are essential for cellular function and survival during apoptosis [56]. For instance, one of the hallmarks of apoptosis is the cleavage of poly ADP-ribose polymerase-1 (PARP-1), a key protein that functions in DNA damage repair [94,95]. During the execution of apoptosis, caspase-3 cleaves PARP-1, releasing a 24 kDa N-terminal fragment that has a DNA binding domain (DBD), which prevents DNA repair during apoptosis [94,95]. Although, caspase-3 is essential for mediating apoptosis, other studies have shown that caspase-3 has additional functions outside of its role in controlling apoptosis. For example, caspase-3 has been identified as a being involved in monocyte-to-macrophage differentiation. When monocytes are stimulated with macrophage colony-stimulating factor (M-CSF) to trigger differentiation into macrophages, active caspase-3 is expressed [96]. Treatment of these cells with the caspase inhibitor z-VAD-fmk blocked monocyte-to-macrophage differentiation [96]. In contrast, no active caspase-3 was detected when monocytes were exposed to dendritic colony-stimulating factors (D-CSF) to trigger differentiation into a dendritic cell [96]. Taken together, these results suggest a role for caspase-3 in shaping differentiation of monocytes specifically toward a macrophage-like phenotype. Our studies have shown that HCMV directly induces the monocyte-to-macrophage differentiation program [47,48,53,54]. We propose that the virus has evolved a mechanism to link survival-to-differentiation in order to generate a long-lived stable cell type that serves as a reservoir for viral dissemination through the regulation of the activity of caspase-3 and the expression of the cellular Bcl-2 family proteins [48]. Early in infection, HCMV inhibits the activation of caspase-3 in order to extend the lifespan of infected monocytes (Figure 2). Viral-induced up-regulation of Mcl-1 prevents the initial cleavage of caspase-3 from its 32-kDa inactive procaspase form into its 20-kDa intermediate form [47]. In addition, HCMV increases the expression of HSP27 to prevent the secondary cleavage of caspase-3 into its 17-kDa fully active form [47]. The fully active 17-kDa form of caspase-3 is only detected at low levels after 48 hpi [47], consistent with a requirement for caspase-3 expression to mediate monocyte-to-macrophage differentiation [96]. In agreement with Sordet et al., our lab demonstrated that when caspase-3 activation is inhibited in HCMV-infected monocytes, the viral-induced differentiation process is abrogated [47]. However, the loss of Mcl-1 and HSP27 results in a robust activation of caspase-3 and high frequency of apoptosis in these infected cells [47]. Taken together, these data suggest that a precise level of caspase-3 activity is required to allow monocyte-to-macrophage differentiation to proceed, while simultaneously preventing the induction of apoptosis. Therefore, we suggest (see Figure 2) that once the infected monocytes survive beyond 48 h viability gate, the virus allows basal activation of the caspase-3 required for differentiation [47,48,52]. Thus, it appears that HCMV utilizes a bi-phasic expression of pro-survival cellular proteins such as Mcl-1 and HSP27 early [47,51], and Bcl-2 late [52], to extend the monocyte lifespan beyond 72 h by regulating caspase-3 activation in a time-dependent manner that coincides with the induction of monocyte-to-macrophage differentiation.

## 3. Viral Proteins/Products

**Figure 3:**
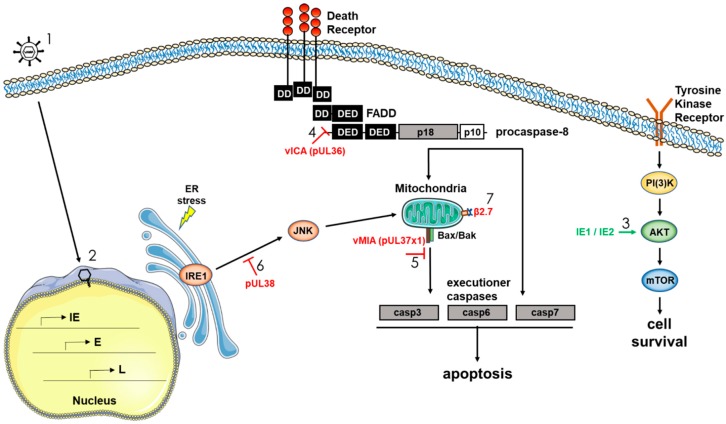
Select HCMV gene products that target apoptotic pathways during HCMV infection. (1) HCMV enters a target cell and (2) the viral capsid is trafficked to the nucleus. The viral genome is translocated into the nuclear space and initiation of viral gene expression occurs. A number of viral gene products have been shown to alter the cellular environment to promote a pro-survival state. (3) The immediate early proteins IE1 and IE2 activate the PI(3)K/Akt/mTOR signaling pathway via phosphorylation of Akt to promote survival of infected cells. (4) The HCMV protein vICA (pUL36) binds to the initiator procaspase-8 and blocks its proteolytic cleavage and activation downstream of cellular death receptors. (5) HCMV vMIA (pUL37x1) binds pro-apoptotic Bax and Bak to prevent mitochondrial outer membrane permeabilization (MOMP) and the induction of the caspase activation cascade. (6) HCMV pUL38 suppresses endoplasmic reticulum (ER) stress-induced apoptosis, likely through inhibition of JNK phosphorylation. Other proteins, as suggested in the text, may also influence survival through a variety of additional mechanisms. (7) In addition to the viral proteins described, HCMV non-coding RNA β2.7 prevents apoptosis via interaction with mitochondrial membrane complex I. FADD: Fas-associated death domain.

HCMV, as a slow replicating virus, must keep infected cells alive for days to weeks to ensure replication and the successful production of new infectious viral particles. The regulation of cellular survival is therefore of critical importance to HCMV persistence, and the virus has been shown to encode a number of gene products that promote survival by targeting multiple host-initiated cell death pathways. As discussed above, we have shown that HCMV initially controls survival of infected monocytes through a distinct regulation of multiple cellular anti-apoptotic factors. Our argument for this reliance on cellular factors early during infection of monocytes stems from the lack of expressed viral gene products in monocytes and macrophages until several weeks after infection [30,48], and that knockdown of these products via siRNA or functional inhibition (or both) through the use of small molecule inhibitors significantly diminishes infected monocyte survival [51,52]. It is important to point out that in our model, we use freshly isolated blood monocytes that are isolated through a gradient and not through positive selection and that are only plated on relevant basement membrane products, thus they only receive adherence to a relevant matrix and HCMV as a stimuli [30,48]. As we have shown, the nature of the stimuli affects the nature of the differentiated cell [52], thus some biological/molecular processes are likely to vary under different physiological conditions. HCMV does encode numerous apoptotic regulators that target key apoptotic steps. In general terms, cell types that are infected and show a classic lytic infection profile (such as endothelial cells, epithelial cells, and fibroblasts) utilize virally encoded anti-apoptotic proteins to block the apoptotic process (reviewed in Reference [97]). In addition, although not formally examined in detail, we expect that in long-term infected macrophages or dendritic cells, many of these virally encoded anti-apoptotic regulators would be able to function in promoting cell survival. Below we briefly review the literature on some of the documented HCMV gene products that specifically block apoptotic pathways (Figure 3).

### 3.1. Viral Inhibitor of Caspase-8-Induced Apoptosis vICA/pUL36

The extrinsic apoptosis pathway can be initiated via intracellular stress signals following ligation/activation of several receptors such as Fas, TRAILR, and TNFR1 [98,99]. The death-inducing signaling complex (DISC), which consists of different binding arrangements among the adapter molecule Fas-associated death domain (FADD), RIP1, cellular FLICE-inhibitory protein (cFLIP), procaspase-8, and/or cellular inhibitor of apoptosis protein (cIAP) is then formed and regulates the initiation of the typical apoptotic pathway. One of the first HCMV proteins identified as having an anti-apoptotic function was HCMV pUL36. The anti-apoptotic function of HCMV pUL36 was found in HeLa cells while screening an HCMV expression library for proteins capable of blocking Fas-induced apoptosis [100]. It was also revealed that pUL36 prevented apoptosis triggered by TNF-α and TRAIL (TNF-related apoptosis inducing ligand) [100]. At early time points, pUL36-deficient mutant viruses were less resistant to Fas- and TNFR-induced apoptosis compared to wildtype virus [100,101]. Mechanistically, pUL36 interacts with procaspase-8 and inhibits its proteolytic processing, thus pUL36 is called the viral inhibitor of caspase-8-induced apoptosis (vICA) [100]. Specifically, vICA interacts with the pro-domain of caspase-8 and inhibits its self-cleavage and maturation [100], presumably by blocking caspase-8 interaction with the adaptor protein FADD [102,103,104]. vICA is conserved in all mammalian betaherpesviruses and has an analogous function in each respective host species [97,103], hinting at an essential role for this protein in the survival of infected cells. The HCMV *UL36* gene maybe dispensable for replication in fibroblasts [105], although this strain of AD169 did have an inactivating UL36 mutation [100]. On the other hand, HCMV vICA seems to be required for efficient replication in differentiated cells of the monocyte-macrophage lineage [101]. Caspase-8 may be critical for monocyte-to-macrophage differentiation [101,106], thus there may be a complex interplay between pUL36 and caspase-8 in long term infection of myeloid cells and HCMV persistence.

### 3.2. Viral Mitochondrial Inhibitor of Apoptosis vMIA/pUL37x1

The UL37 protein exists in several isoforms: the full-size gpUL37, the UL37 exon 1 protein (pUL37x1), and the UL37 medium protein (pUL37-M) (reviewed in References [107,108]). pUL37x1 is known as viral mitochondria-localized inhibitor of apoptosis (vMIA) and is the predominant UL37 product produced during permissive HCMV infection [109]. Full size gpUL37 protein is expressed at very low levels and pUL37-M has not yet been detected in infected cells. The vMIA protein is localized to the mitochondrial compartment [109]. At the biochemical level, vMIA blocks mitochondrial release of cytochrome C by preventing permeabilization of the mitochondria outer membrane, and blocks procaspase-9 maturation to active caspase-9 [109,110,111,112,113,114,115]. It also strongly inhibits ER stress apoptosis [112]. The molecular mechanism of vMIA anti-apoptotic activity is believed to be its ability to interact with Bax or Bak (or both) to prevent its pro-apoptotic function at the mitochondria [97,109,116,117]. Besides blocking caspase-dependent apoptosis, vMIA can also control a caspase-independent cell death pathway initiated by the mitochondrial serine protease HtrA2/Omi [118]. The murine cytomegalovirus proteins m38.5 (vMIA) and m41.1 (viral inhibitor of Bak oligomerization; vIBO) function analogously by binding Bak or Bax (or both), highlighting the importance of this virally-encoded anti-apoptotic protein to survival of infected cells [115,117,119,120,121].

### 3.3. HCMV UL38 Protein

There are two reported activities attributed to the HCMV UL38 encoded protein: an anti-apoptotic activity and an activation of protein synthesis, which improves cell viability. For example, it has been shown that the HCMV *UL38* gene product encodes an ER-located cell death inhibitory protein that can prevent proteolysis of two key apoptotic enzymes, caspase-3 and poly(ADP-ribose) polymerase [122], perhaps via inhibition of persistent JNK phosphorylation [123]. Independent of its ability to prevent ER-stress induced cell death, pUL38 also can regulate protein synthesis. That is, UL38 can affect the accumulation of ATF4 (activating transcriptional factor 4), a transcription factor that can alter the activation of the ER-stress sensor PERK (protein kinase R-like ER kinase) and one of the mTOR complexes, mTORC1 [123,124].

### 3.4. HCMV Major Immediate-Early Proteins IE1 and IE2

HCMV encodes two major immediate-early (mIE) proteins, which arise from the major IE promoter (MIEP) as a result of alternative splicing and polyadenylation of the primary transcript. Proteins are subdivided into what is considered classic IE1 (containing exon 4 sequences) and IE2 (containing exon 5 sequences) proteins. IE1, a 72 kDa protein (also called IEP72 or IE72) is a product of the *UL123* gene; and IE2 (86 kDa; also called IEP86 or IE86) is a product of the *UL122* gene [125]. They share 85 amino-terminal residues corresponding to exons 2 and 3, but have a different carboxy-terminus; encoded by exon 4 (IE1) or exon 5 (IE2) [126]. IE2 is indispensable for productive viral replication, while IE1 is conditionally essential [126]. The mechanisms for anti-apoptotic activity of the major IE proteins [127] are not completely understood. IE2 can upregulate retinal c-FLIP, a protease-deficient procaspase-8 homologue, and decrease the activities of caspases 3 and 8 [128]. IE1 and IE2 do not appear to interfere with mitochondria-related apoptotic processes [127], however, they can activate the PI3K-Akt-mTOR pro-survival pathway [129,130,131]. The major IE proteins can also influence host gene expression and dysregulate multiple signaling pathways [132,133,134]. Thus, IE proteins play a very sophisticated role in regulation of protein synthesis and thus likely control the process of apoptosis through multiple mechanisms.

### 3.5. HCMV β2.7 RNA

Another viral gene product that promotes survival of infected cells is HCMV β2.7. Although β2.7 does not encode a protein, the gene gives rise to a 2.7 kb un-spliced polyadenylated RNA and is the most abundantly transcribed early gene in permissive cells [135,136]. Peak expression of HCMV β2.7 occurs between 8 and 14 hpi, and the gene remains transcriptionally active throughout the viral replication cycle [137]. It is unclear whether the gene is expressed during a latent or otherwise quiescent infection such as would occur in monocytes and other myeloid cells [25,138]; however, it is likely that β2.7 would be expressed abundantly following monocyte-to-macrophage differentiation and viral reactivation. A study by Reeves et al. showed that β2.7 interacts with the mitochondrial enzyme complex I to stabilize mitochondrial membrane potential and prevent apoptotic death of HCMV-infected neuronal cells [139]. β2.7 does this by directly binding genes associated with retinoid/interferon-induced mortality 19, a subunit of the mitochondrial enzyme complex I. This interaction also results in continued adenosine triphosphate production, which is required for HCMV to successfully complete its viral life cycle. Overexpression of β2.7 in rat aortic endothelial cells has also been shown to protect against apoptosis during ischemia/reperfusion injury by reducing the production of reactive oxygen species [140].

## 4. Summary/Discussion

Successful viral replication relies on evasion of the host defense mechanism that limits viral replication by killing infected cells. HCMV encodes a number of viral proteins that promote survival of replication-permissive cells by inhibiting or delaying apoptosis until sufficient viral replication has occurred. In non-permissive cells (such as in monocytes or in any cell that is initially non-permissive), the virus has evolved a sophisticated mechanism to usurp cellular signaling pathways and alter the expression of cellular anti-apoptotic proteins. Importantly, HCMV utilizes these proteins in a manner that appears distinct from that seen in mock-infected cells or in cells treated with other stimuli (e.g., LPS). In addition, HCMV, like other herpesviruses, employs a variety of strategies to subvert alternative mechanisms that promote cell death (e.g., necroptosis) [141,142,143,144,145]. It is clear that the mechanisms by which HCMV promotes survival of infected cells are complex and multi-faceted. Additional work will be needed in the future to fully understand the mechanisms by which HCMV promotes survival of infected cells and to identify therapeutic targets that could potentially limit viral replication and spread in the host.
